# Genetic variations in the TERT and CLPTM1L gene region and gastrointestinal stromal tumors risk

**DOI:** 10.18632/oncotarget.5153

**Published:** 2015-09-08

**Authors:** Rui Zhang, Jian Zhao, Jian Xu, Fang Liu, Yongqing Xu, Xianmin Bu, Chaoliu Dai, Chun Song

**Affiliations:** ^1^ Department of Colorectal Surgery, Liaoning Cancer Hospital & Institute, Shenyang 110042, Liaoning Province, P.R. China; ^2^ Department of Hepatobiliary and Splenic Surgery, Shengjing Hospital, China Medical University, Shenyang 110004, Liaoning Province, P.R. China

**Keywords:** gastrointestinal stromal tumors, GIST, TERT, CLPTM1L, polymorphism

## Abstract

Recent studies have suggested polymorphisms in the TERT and CLPTM1L region are associated with carcinogenesis of many distinct cancer types, including gastrointestinal cancers. However, the contribution of polymorphisms in the TERT and CLPTM1L gene region to gastrointestinal stromal tumors (GISTs) risk is still unknown. We tested the six tagSNPs on TERT and CLPTM1L region with GIST risk, using a population-based, two-stage, case-control study in 2,000 subjects. Functional validation was conducted to validate our findings of TERT rs2736098 and explore its influence on relative telomere length (RTL) in GIST cells. It showed that variant rs2736098 was significantly associated with increased risk of GIST (per allele OR = 1.29, 95% CI: 1.14–1.47, *P* = 7.03 × 10^−5^). The difference remain significant after Bonferroni correction (*P* = 7.03 × 10^−5^ * 6 = 4.2 × 10^−4^). Real-time PCR showed carriers of genotype CC have the longest RTL, following by carriers of genotype CT, while carriers of genotype TT have the shortest RTL in GIST tissues (*P* < 0.001). Our data provide evidence to implicate TERT rs2736098 polymorphism as a novel susceptibility factor for GIST risk.

## INTRODUCTION

Gastrointestinal stromal tumors (GISTs) are the most common mesenchymal tumors in the human digestive tract, representing 1–3% of gastrointestinal malignancies [1, 2]. The histogenesis, classification, diagnostic criteria, and biological behavior of GISTs have been the subject of much controversy [2]. Simply, they are typically defined as tumors whose behavior is driven by mutations in the Kit gene or PDGFRA gene [3–5]. The mechanism of activation in part of sporadic GISTs is an alteration of the structure of the receptor's extracellular or cytoplasmic domains caused by somatic mutations of the c-kit gene, which leads to dimerization and autophosphorylation of KIT with subsequent activation of signal transduction cascades in the absence of ligand binding [6–8]. Inhibition of KIT activity by a specific tyrosine kinase inhibitor, imatinib, often results in dramatic clinical responses [8, 9].

In contrast to GISTs associated with somatic mutations, little is known about inherited germline genetic risk factors. The rarity of the disease makes it a difficult subject to conduct population-based genetic research and unbiased assessment of non-genetic risk factors in any study population. An evaluation of the genetic determinants of GISTs is much more feasible, as the germline DNA of individuals does not change over time or in response to disease processes. Recently, O'Brien et al [10] only evaluated the associations between some candidate SNPs and several common types of acquired KIT and PDGFRA somatic mutations in a case-only study for the first time. However, no other research groups have published such evaluations, not to mention germline genetic associations with GISTs.

The TERT and CLPTM1L gene have been identified to be associated with carcinogenesis of at least 15 distinct cancers [11–14]. TERT promoter mutations were also detected in GIST tissues [15]. Recently, six tagSNPs on TERT and CLPTM1L region (five SNPs in the TERT gene: rs7726159, rs2853677, rs2736098, rs13172201, rs10069690; one SNP in CLPTM1L gene: rs451360) were identified, all of which influenced the risk of multiple cancers, including kinds of gastrointestinal cancers [16]. Given this evidence, our main objective was to identify whether these six tagSNPs potentially related to GIST carcinogenesis. Therefore, we first conducted this large population-based, two-stage, case-control study of GIST risk.

## RESULTS

A total of 2,000 subjects were included in the current study; 600 were genotyped in Stage I and 1,400 were genotyped in Stage II (Table 1). People in the two genotyping stages were generally comparable. As expected, GIST cases were found to differ from controls in regard to known cancer risk factors: cases were more likely to have a higher education, body mass index (BMI), waist-to-hip ratio (WHR), and more likely to smokers and drinkers. Most of the GISTs were located in stomach (63.8%) or small Intestine (32.5%).

**Table 1:** Characteristics of the study population

Characteristics	Stage I (*N* = 600)			Stage II (*N* = 1,400)		
	Cases (*n* = 300)	Controls (*n* = 300)	*P* value	Cases (*n* = 700)	Controls (*n* = 700)	*P* value
Age (years)	50.0 ± 4.39	50.5 ± 4.19	0.193	50.2 ± 3.1	50.1 ± 3.1	0.103
Gender (male)	234 (78.0%)	219 (73.0%)	0.154	515(73.6%)	514 (73.4%)	0.952
Education (less than middle school)	53(17.7%)	34 (11.3%)	**0.028**	87(12.4%)	86(12.6%)	0.998
Body mass index (kg/m^2^)	24.1 ± 2.0	23.9 ± 2.22	0.513	24.3 ± 2.4	24.0 ± 2.3	**0.008**
Waist-to-hip ratio	0.8223 ± 0.004	0.8200 ± 0.004	**<0.0001**	0.8223 ± 0.004	0.8199 ± 0.003	**<0.0001**
Regular physical activity	83(27.7%)	99 (33.0%)	0.155	181(25.9%)	195 (27.9%)	0.399
Ever smokers	81(27.0%)	64(21.3%)	0.105	201(28.7%)	110(15.7%)	**<0.0001**
Ever drinkers	88(29.3%)	50(16.7%)	**<0.0001**	188(26.9%)	153(21.9%)	**0.029**
**Tumor Location**						
Stomach	191(63.6%)			448(64.0%)		
Small Intestine	96(32.0%)			228 (32.6%)		
Rectum	5 (1.6%)			9 (1.3%)		
Other	8 (2.6%)			15 (2.1%)		

Continuous variables: mean values ± standard deviation, *p*-value from *t*-tests;

Categorical variables: numbers and percentages, *p*-values from *x*^2^test

*P* value in bold means statistically significant.

A total of six tagSNPs on TERT and CLPTM1L region were included in the current study; of these, five SNPs were located in the TERT gene (rs7726159, rs2853677, rs2736098, rs13172201, rs10069690) and one SNP was located in neighboring CLPTM1L gene (rs451360). None of the six polymorphisms were found to deviate from HWE. We first evaluated the six tagSNPs in Stage I with 300 cases and 300 controls. The estimates of effect on GIST risk in Stage I, adjusted for age and gender are shown in Table 2. Three SNPs (rs7726159, rs10069690, and rs2736098) were found to have associations of significance with GIST risk. Then they were evaluated in Stage II with 700 cases and 700 controls additionally (Table 3). One SNP (rs2736098) was replicated with significance (*P* = **7.03 × 10^−5^**). The difference remain significant after Bonferroni correction (*P* = **7.03 × 10^−5^** * 6 = 4.2 × 10^−4^). Compared with individuals with the CC genotype, the age and sex adjusted OR for developing GIST was 1.49 (95% CI 1.18–1.88) among those with the TT genotype. Under the log-additive model, each additional copy of minor allele A was associated with a 1.29-fold increased risk of GIST (OR = 1.29, 95% CI: 1.14–1.47, *P* = **7.03 × 10^−5^**).

**Table 2:** Association between tagSNPs on TERT and CLPTM1L region and GIST risk (Stage I)

SNP	Alleles^a^	MAF^b^	AB OR (95% CI)	BB OR (95% CI)	B vs A OR (95% CI)	*P*
rs7726159	C/A	0.33	1.32 (0.93–1.87)	1.67 (1.04–2.68)	1.33 (1.05–1.69)	**0.017**
rs2853677	A/G	0.41	0.94 (0.66–1.34)	1.72 (0.41–1.24)	0.89 (0.71–1.13)	0.894
rs2736098	C/T	0.37	1.49 (1.03–2.14)	1.78 (1.17–2.72)	1.44 (1.14–1.81)	**1.92 × 10^−3^**
rs13172201	C/T	0.25	1.18 (0.82–1.69)	1.24 (0.75–2.05)	1.17 (0.90–1.50)	0.241
rs10069690	C/T	0.16	1.36 (0.96–1.93)	2.82 (0.91–8.74)	1.40 (1.04–1.88)	**0.025**
rs451360	C/A	0.14	1.13 (0.76–1.69)	1.52 (0.69–3.33)	1.22 (0.89–1.68)	0.222

a Major/minor alleles as determined by allele frequency among genotyped controls

b Minor allele frequency among genotyped controls

AA: major allele homozygotes (reference group); AB: heterozygotes; BB: minor allele homozygotes; A: major allele; B: minor allele

*P* value in bold means statistically significant.

**Table 3:** Association between tagSNPs on TERT and CLPTM1L region and GIST risk (Stage II)

SNP	Alleles^a^	Stage	AB OR (95% CI)	BB OR (95% CI)	B vs A OR (95% CI)	*P*
rs7726159	C/A	I	1.32 (0.93–1.87)	1.67 (1.04–2.68)	1.33 (1.05–1.69)	**0.017**
		II	0.94 (0.75–1.19)	0.95 (0.71–1.29)	0.96 (0.82–1.13)	0.630
rs10069690	C/T	I	1.36 (0.96–1.93)	2.82 (0.91–8.74)	1.40 (1.04–1.88)	**0.025**
		II	0.92 (0.74–1.15)	0.83 (0.27–2.49)	0.93 (0.77–1.13)	0.489
rs2736098	C/T	I	1.49 (1.03–2.14)	1.78 (1.17–2.72)	1.44 (1.14–1.81)	**1.92 × 10^−3^**
		II	1.27 (1.03–1.60)	1.38 (1.04–1.83)	1.23 (1.06–1.44)	**6.63 × 10^−3^**
		Combined	1.33 (1.09–1.101.62)	1.49 (1.18–1.88)	1.29 (1.14–1.47)	**7.03 × 10^−5^**

a Major/minor alleles as determined by allele frequency among genotyped controls

AA: major allele homozygotes (reference group), AB: heterozygotes, BB: minor allele homozygotes;

*P* value in bold means statistically significant.

**Figure 1:**
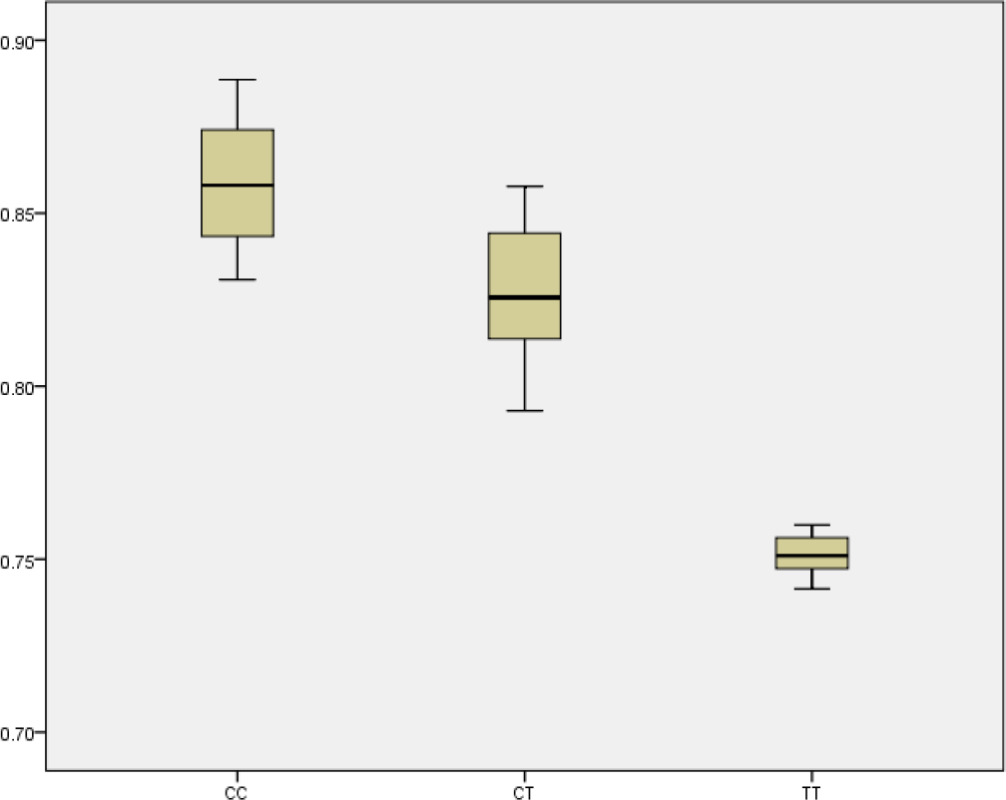
Boxplot for the RTL with different genotype of SNP rs2736098

The robustness of these findings was evaluated by sensitivity analyses. First, additional adjustments by education, BMI, WHR, physical activity, drinking and smoking were conducted respectively. The results didn't change materially. To validate our findings of TERT rs2736098 and explore its influence on RTL, we used real-time PCR to measure the RTL in a random sample of 150 GIST cases. In GIST tissues, We found a significant difference in RTLs among the genotype CC, CT, and TT, respectively (Figure 1, *P* < 0.001).

## DISCUSSION

To the best of our knowledge, this is the first report to attempt an evaluation of the association of six tagSNPs on TERT and CLPTM1L region potentially related to GIST carcinogenesis. In this large population-based, two-stage, case-control study, we identified that the variant rs2736098 was significantly associated with increased risk of GIST, especially for GISTs located in stomach. To validate this finding, real-time PCR showed that the RTL in GIST cells were significantly lower than that of their adjacent normal tissues. And in GIST tissues, carriers of genotype CC have the longest RTL, following by carriers of genotype CT, while carriers of genotype TT have the shortest RTL. These provide evidence to implicate rs2736098 polymorphism as a novel susceptibility factor for GIST risk.

GISTs are the most common soft tissue sarcoma of the gastrointestinal tract, resulting most commonly from KIT or platelet-derived growth factor receptor alpha (PDGFRalpha)-activating mutations [19–21]. However, they have distinct genetic background and gene expression patterns according to localization, genotype and aggressiveness [22, 23]. Chr5p15.33 harbors a unique cancer susceptibility region that contains at least two plausible candidate genes: TERT and CLTPM1L [24–27]. The TERT gene has been mapped to chromosome 5p15.33 and consisted of 16 exons and 15 introns spanning 35kb of genomic DNA [28]. It encodes the catalytic subunit of the telomerase reverse transcriptase, which, in combination with an RNA template (TERC), adds nucleotide repeats to chromosome ends [29, 30]. The CLTPM1L gene, also known as cisplatin resistance-related protein 9 (CRR9p), encodes a protein that is overexpressed in lung and pancreatic cancer, promotes growth and survival, and is required for KRAS driven lung cancer [31, 32]. It confer resistance to apoptosis caused by genotoxic agents in association with up-regulation of the anti-apoptotic protein, Bcl-xL [33]. Studies indicate that the TERT-CLPTM1L region may harbor multiple elements that have the capacity to influence molecular phenotypes in cancer development [16, 34]. Thus, It is possible to study that the interplay between risk variants, multiple biological mechanisms and attributed genes, influence various cancers, including GISTs.

Although few literature investigating the associations somatic mutations of TERT and GIST risk [15, 35, 36], none has evaluated germline genetic associations with GISTs. In current study, we identified rs2736098 contribute to increased risk of GIST and shorter RTL, using a two-stage, case-control study. This finding is consistent with many previous epidemiological studies with different cancer types, including lung cancer, bladder cancer, pancreatic cancer, gastrointestinal cancers, breast cancer, ovarian cancer, and so on [16, 37–39]. All of evidence above implicate TERT rs2736098 polymorphism as a novel susceptibility factor for carcinogenesis.

Considering rs2736098 polymorphism being a tagSNP, it is possible the association seen with rs2736098 tagSNP is due to one of those linked polymorphisms. We additionally listed the detailed information for these 11 linked polymorphisms of rs2736098 in Table 4. Among them, 9 are intergenic SNPs, and 2 are intron variants. Although rs2736098 was a synonymous SNP, our results showed that carriers of genotype CC have the longest RTL, following by carriers of genotype CT, while carriers of genotype TT have the shortest RTL in GIST tissues (*P* < 0.001). This evidence yet indicate the functionality of SNP rs2736098. Further fine mapping and sequencing studies may be helpful for the validation the our conclusions.

**Table 4:** Detailed information for linked SNPs of rs2736098

SNP	Gene	Alleles^a^	Positions	Region/Functionality
**rs2736098**	**TERT**	**C/T**	**chr5:1293836**	**Exon/synonymous**
rs2736109	**TERT**	A/G	chr5:1296759	intergenic
rs2736108	**TERT**	G/A	chr5: 1297488	intergenic
rs2853672	**TERT**	C/A	chr5: 1293233	intron
rs2735940	**TERT**	G/A	chr5: 1296736	intergenic
rs2736103	**TERT**	A/G	chr5: 1300401	intergenic
rs2735846	**TERT**	C/G	chr5: 1299379	intergenic
rs13174919	**TERT**	C/G	chr5: 1300112	intergenic
rs4975612	**TERT**	G/T	chr5: 1300310	intergenic
rs13174814	**TERT**	C/G	chr5: 1300109	intergenic
rs2736099	**TERT**	C/T	chr5: 1287340	intron
rs2736105	**TERT**	A/G	chr5: 1299756	intergenic

a Major/minor alleles as determined by allele frequency among genotyped controls

Strengths of the current study include a large population, a two-stage genotyping design to minimize type I error, and good coverage of the genetic variation in the TERT and CLPTM1L region. This study also had several limitations. First, selection bias might have occurred through the selection of control subjects when the sampling is not random within the subpopulations of cancer and cancer-free subjects, though we have try our best to control it through the whole process of the study; since this study was restricted to a Chinese Han population, it is uncertain whether our findings can be replicated by other ethnic groups. Second, in spite of the relatively large sample size, the power to elucidate gene–environment interactions was limited because of the small magnitude of the overall association.

In summary, our findings regarding genetic variation in the TERT and CLPTM1L region and GIST risk add to the growing body of literature suggesting the importance of this genetic region to cancer development. Further research is needed in this area to understand how changes in telomere length over time may influence GIST carcinogenesis in a prospective setting and interaction with a p53 pathway of development.

## MATERIALS AND METHODS

### Subjects

The methods were carried out in “accordance” with the approved guidelines. Also, all experimental protocols were approved by the institutional review boards of liaoning cancer hospital and shengjing hospital, and written informed consent was obtained from all participants. Cases were histopathologically confirmed GIST patients. Controls without clinic evidence of gastrointestinal diseases or tumors were randomly selected from a pool of healthy volunteers who visited the general health checkup center of the same hospital for routine scheduled physical exams. Controls were individually matched to cases on sex, ethnicity (Han), age (±5 years). After giving written consent, participants provided demographic information using a standard interviewer-administered questionnaire. Stage I includes 300 GIST cases and 300 controls, while stage II includes 700 GIST cases and 700 controls. Totally incluede in this study were 1,000 cases and 1,000 controls. Five ml of peripheral blood was obtained for DNA extraction.

### SNP selection and genotyping

Totally, five SNPs in the TERT gene (rs7726159, rs2853677, rs2736098, rs13172201, rs10069690) and one SNP in neighboring CLPTM1L gene (rs451360) were selected in this study (details in Table 5 and supplementary table 1), according to the previous literature [16]. 5′-Nuclease TaqMan^®^ assays were used to genotype the polymorphisms in 96-well plates on an ABI PRISM 7900HT Sequence Detection system (Applied BioSystems, Foster City, CA, USA). The primers and probes for the TaqMan^®^ assays were designed using Primer Express Oligo Design software v2.0 (ABI PRISM) and are available upon request as TaqMan^®^ Pre-Designed SNP Genotyping Assays. Samples from matched case-control pairs were handled identically and genotyped in the same batch in a blinded fashion. All included SNPs had concordance rates of 100% among duplicates within each platform, and laboratory personnel were blinded to the case–control and QC status of all samples.

**Table 5:** Basic information for each tagSNP

SNP	Gene	Alleles^a^	Positions	Region/Functionality
rs7726159	TERT	C/A	chr5:1282069	intron
rs2853677	TERT	A/G	chr5:1286944	intron
rs2736098	TERT	C/T	chr5:1293836	Exon/synonymous
rs13172201	TERT	C/T	chr5:1269156	intron
rs10069690	TERT	C/T	chr5:1279540	intron
rs451360	CLPTM1L	C/A	chr5:1319680	intron

a Major/minor alleles as determined by allele frequency among genotyped controls

### Relative telomere length (RTL) determination

RTL of GIST cells were measured using quantitative real-time polymerase chain reaction (PCR) as described earlier in a random sample of 150 GIST cases [17]. In short, telomeres and a single-copy gene (β2-globin) were amplified including an internal reference control cell line (CCRF-CEM) to which all samples were compared. The ΔΔCt method was used for calculation of RTL values and a standard curve was created in each PCR run to monitor the PCR efficiency.

### Statistical Analyses

Hardy–Weinberg equilibrium (HWE) was tested by comparing observed and expected genotype frequencies among controls (x^2^ test). Odds ratios (ORs) and corresponding 95% confidence intervals (CIs) were determined by logistic regression analyses using models that included adjustment for age and gender. Linkage disequilibrium (LD) was assessed by Haploview [18]. Differences of RTLs among different groups were compared by One-Way ANOVA method. All statistical analyses were conducted with SAS version 9.2 (SAS Institute Inc.). All statistical tests were 2-tailed, and *P* < 0.05 was interpreted as statistically significant unless otherwise indicated.

## SUPPLEMENTARY TABLE


