# Supplementary material for: Genetic variations in the TERT and CLPTM1L gene region and gastrointestinal stromal tumors risk
Source: Oncotarget. 2015 Sep 8;6(31):31360–7. doi: 10.18632/oncotarget.5153 (PMC4741611; doi:10.18632/oncotarget.5153)
Supplement: Supplementary file 1 [file oncotarget-06-31360-s001.pdf]

SUPPLEMENTARY TABLES

Supplementary Table S1: Variants represented by each tagSNP

SNP	Proxy	Distance	RSquared	DPrime	Chromosome	Coordinate_HG18
rs7726159	rs2736100	4197	0.828	0.926	chr5	1339516
rs7726159	rs7725218	95	0.759	0.887	chr5	1335414
rs7726159	rs7734992	2191	0.692	0.847	chr5	1333128
rs7726159	rs4975538	1489	0.662	0.874	chr5	1333830
rs7726159	rs10054203	2355	0.66	0.812	chr5	1332964
rs7726159	rs35888851	785	0.618	0.872	chr5	1336104
rs7726159	rs2853677	4875	0.545	0.855	chr5	1340194
rs7726159	rs2736099	5021	0.474	0.74	chr5	1340340
rs7726159	rs72709458	1436	0.329	0.839	chr5	1336755
rs7726159	rs10069690	2529	0.329	0.839	chr5	1332790
rs2853677	rs2736099	146	0.719	0.914	chr5	1340340
rs2853677	rs2736100	678	0.586	0.902	chr5	1339516
rs2853677	rs7726159	4875	0.545	0.855	chr5	1335319
rs2853677	rs2853672	5789	0.543	0.947	chr5	1345983
rs2853677	rs2735940	9292	0.524	0.946	chr5	1349486
rs2853677	rs7725218	4780	0.447	0.761	chr5	1335414
rs2853677	rs7734992	7066	0.441	0.755	chr5	1333128
rs2853677	rs35888851	4090	0.42	0.832	chr5	1336104
rs2853677	rs10054203	7230	0.417	0.748	chr5	1332964
rs2853677	rs4975538	6364	0.335	0.624	chr5	1333830
rs2853677	rs13167280	6717	0.308	1	chr5	1333477
rs2736098	rs2736109	2673	0.888	0.96	chr5	1349759
rs2736098	rs2736108	3402	0.723	1	chr5	1350488
rs2736098	rs2853672	1103	0.63	1	chr5	1345983
rs2736098	rs2735940	2400	0.609	1	chr5	1349486
rs2736098	rs2736103	6315	0.449	0.735	chr5	1353401
rs2736098	rs2735846	5293	0.426	0.751	chr5	1352379
rs2736098	rs13174919	5776	0.425	0.767	chr5	1352862
rs2736098	rs4975612	6224	0.397	0.835	chr5	1353310
rs2736098	rs13174814	5773	0.388	0.759	chr5	1352859
rs2736098	rs2736099	6746	0.384	0.655	chr5	1340340
rs2736098	rs2736105	5670	0.344	0.634	chr5	1352756
rs13172201	rs11133719	137	0.459	0.722	chr5	1324524
rs10069690	rs72709458	3965	1	1	chr5	1336755

(*Continued*)

SNP	Proxy	Distance	RSquared	DPrime	Chromosome	Coordinate_HG18
rs10069690	rs2242652	238	0.817	1	chr5	1333028
rs10069690	rs56345976	2917	0.559	1	chr5	1329873
rs10069690	rs4975538	1040	0.539	1	chr5	1333830
rs10069690	rs7734992	338	0.484	1	chr5	1333128
rs10069690	rs10054203	174	0.467	1	chr5	1332964
rs10069690	rs7725218	2624	0.414	0.925	chr5	1335414
rs10069690	rs2736100	6726	0.381	0.919	chr5	1339516
rs10069690	rs7726159	2529	0.329	0.839	chr5	1335319
rs10069690	rs73023637	473618	0.313	1	chr5	859172
rs451360	rs13170453	2199	1	1	chr5	1370481
rs451360	rs36115365	6438	1	1	chr5	1366242
rs451360	rs35953391	7351	1	1	chr5	1365329
rs451360	rs380145	9217	1	1	chr5	1381897
rs451360	rs4635969	11128	1	1	chr5	1361552
rs451360	rs7446461	13159	1	1	chr5	1359521
rs451360	rs27071	26401	1	1	chr5	1399081
rs451360	rs27068	27559	1	1	chr5	1400239
rs451360	rs37004	37004	0.925	1	chr5	1409684
rs451360	rs71595005	18843	0.85	1	chr5	1353837
rs451360	rs111986123	22477	0.81	1	chr5	1395157
rs451360	rs37002	37264	0.733	0.92	chr5	1409944
rs451360	rs421629	456	0.714	1	chr5	1373136
rs451360	rs381949	2788	0.714	1	chr5	1375468
rs451360	rs13178866	3532	0.714	1	chr5	1376212
rs451360	rs421284	5910	0.714	1	chr5	1378590
rs451360	rs466502	6087	0.714	1	chr5	1378767
rs451360	rs465498	6123	0.714	1	chr5	1378803
rs451360	rs452932	10573	0.714	1	chr5	1383253
rs451360	rs452384	11160	0.714	1	chr5	1383840
rs451360	rs370348	11539	0.714	1	chr5	1384219
rs451360	rs457130	16498	0.714	1	chr5	1389178
rs451360	rs467095	16541	0.714	1	chr5	1389221
rs451360	rs455433	16563	0.714	1	chr5	1389243
rs451360	rs460073	16779	0.714	1	chr5	1389459
rs451360	rs462608	16946	0.714	1	chr5	1389626
rs451360	rs456366	17390	0.714	1	chr5	1390070
rs451360	rs459961	17426	0.714	1	chr5	1390106

(*Continued*)

SNP	Proxy	Distance	RSquared	DPrime	Chromosome	Coordinate_HG18
rs451360	rs31484	18226	0.714	1	chr5	1390906
rs451360	rs31489	23034	0.714	1	chr5	1395714
rs451360	rs31490	24778	0.714	1	chr5	1397458
rs451360	rs27996	25794	0.714	1	chr5	1398474
rs451360	rs27070	26623	0.714	1	chr5	1399303
rs451360	rs37011	29118	0.714	1	chr5	1401798
rs451360	rs40181	34782	0.714	1	chr5	1407462
rs451360	rs76236979	15379	0.706	1	chr5	1357301
rs451360	rs3816659	1860	0.673	1	chr5	1370820
rs451360	rs4975616	4020	0.673	1	chr5	1368660
rs451360	rs4975615	4337	0.673	1	chr5	1368343
rs451360	rs10078017	5671	0.673	1	chr5	1367009
rs451360	rs28379291	5979	0.673	1	chr5	1366701
rs451360	rs13356727	7223	0.673	1	chr5	1365457
rs451360	rs6866783	7660	0.673	1	chr5	1365020
rs451360	rs6866294	7987	0.673	1	chr5	1364693
rs451360	rs11133727	12915	0.673	1	chr5	1359765
rs451360	rs4404721	13515	0.673	1	chr5	1359165
rs451360	rs2735945	15779	0.673	1	chr5	1356901
rs451360	rs2853666	16766	0.673	1	chr5	1355914
rs451360	rs117256253	26872	0.673	1	chr5	1399552
rs451360	rs414965	4441	0.638	0.917	chr5	1377121
rs451360	rs37010	29855	0.638	0.917	chr5	1402535
rs451360	rs37009	30659	0.638	0.917	chr5	1403339
rs451360	rs40182	30717	0.638	0.917	chr5	1403397
rs451360	rs37008	31858	0.638	0.917	chr5	1404538
rs451360	rs37007	32692	0.638	0.917	chr5	1405372
rs451360	rs37006	35378	0.638	0.917	chr5	1408058
rs451360	rs37003	37091	0.638	0.917	chr5	1409771
rs451360	rs55901723	22474	0.636	1	chr5	1395154
rs451360	rs35029535	34704	0.636	1	chr5	1337976
rs451360	rs4530805	13349	0.602	1	chr5	1359331
rs451360	rs2447853	13397	0.602	1	chr5	1386077
rs451360	rs6554758	9528	0.598	0.915	chr5	1363152
rs451360	rs27069	27448	0.598	0.915	chr5	1400128
rs451360	rs37005	36770	0.494	0.755	chr5	1409450
rs451360	rs61574973	10512	0.456	0.75	chr5	1362168

(*Continued*)

SNP	Proxy	Distance	RSquared	DPrime	Chromosome	Coordinate_HG18
rs451360	rs6890396	39418	0.368	1	chr5	1412098
rs451360	rs33963617	40241	0.368	1	chr5	1332439
rs451360	rs117468417	2891	0.362	0.754	chr5	1369789
rs451360	rs402710	1042	0.347	1	chr5	1373722
rs451360	rs401681	2407	0.333	1	chr5	1375087
